# Minimally invasive versus conventional apical enlargement during retreatment of severely curved mesial canals: A micro–CT study

**DOI:** 10.1007/s00784-026-06960-2

**Published:** 2026-06-05

**Authors:** Poliana J. P. da Silva, Marília F. V. Marceliano-Alves, Lúcio Sousa Gonçalves, Flávio R. F. Alves, José C. Provenzano

**Affiliations:** 1 Postgraduate Program in Dentistry, Estácio de Sá University, Rio de Janeiro, RJ Brazil; 2 Department of Endodontics, Faculty of Dentistry, Iguaçu University (UNIG), Nova Iguaçu, RJ Brazil; 3 Postgraduate Program in Dentistry, University of Grande Rio (UNIGRANRIO), Rio de Janeiro, RJ Brazil; 4 Rua Professor José de Souza Herdy, 1160, Duque de Caxias, RJ 25071-202 Brazil

**Keywords:** Endodontic retreatment, D-Race, Filling removal, Micro-computed tomography, Mtwo, Nickel-titanium instruments.

## Abstract

**Objectives:**

This study used micro–computed tomography (micro-CT) to compare a minimally invasive retreatment approach (reinstrumentation 30/0.04) with a conventional retreatment protocol (reinstrumentation 40/0.04) during different stages of filling removal in curved mesial roots of mandibular molars.

**Materials and methods:**

Fourteen extracted mandibular first molars with Vertucci type IV and severe curvature (25°–40°) were selected, endodontically prepared (25/0.04), and obturated. A paired intra-root design compared minimally invasive retreatment (D-Race + iRace to 30/0.04) with a conventional protocol (Mtwo to 40/0.04). Retreatments were performed without solvents, and micro-CT scans were taken at three stages. Residual filling material was quantified and analyzed at a 5% significance level.

**Results:**

Initial filling volumes were similar between protocols (*P* > .05). After desobstruction, the minimally invasive protocol removed more material (*P* < .01), leaving 0.2 mm³ (95%) versus 1.1 mm³ (70%). However, after reinstrumentation, no significant differences were observed (*P* > .05), with both protocols achieving high removal rates (98% vs. 95%).

**Conclusion:**

The minimally invasive protocol showed greater efficiency in the initial removal of obturation material. After final reinstrumentation, both approaches achieved similar outcomes, with < 5% residual material. These findings suggest that conservative enlargement to 30/0.04 may be as effective as 40/0.04 while better preserving radicular dentin.

**Clinical relevance:**

Extensive apical enlargement (40/0.04) is not essential for effective removal of obturation material in severely curved canals. A conservative approach (30/0.04) achieves similar cleanliness while better preserving radicular dentin and structural integrity.

## Introduction

The long-term success of endodontic treatment primarily depends on the effective elimination and control of intraradicular infection [1]. However, technical and biological limitations may compromise complete disinfection, allowing microorganisms to persist and potentially leading to treatment failure. This often results in post-treatment apical periodontitis, even in teeth that appear radiographically well-filled [2, 3]. In such cases, nonsurgical endodontic retreatment is indicated to remove existing filling material, reestablish access to the full canal system, and create conditions favorable for periradicular healing [4].

A critical step in retreatment is the effective removal of filling materials, as remnants of gutta-percha and sealer may harbor microorganisms and prevent adequate disinfection [5]. Nevertheless, complete removal of obturation material remains challenging due to the anatomical complexity of the root canals, the compaction and penetration of filling materials into irregularities, and the physical properties of the materials themselves [5]. Previous evidence suggests that greater removal of filling material is associated with improved retreatment outcomes [5].

Nickel–titanium rotary systems specifically designed for retreatment have been developed to improve the efficiency and safety of filling material removal [6]. Among the well-established systems in clinical practice are the D-Race/iRace (FKG Dentaire, La Chaux-de-Fonds, Switzerland) and Mtwo Retreatment/Mtwo (VDW, Munich, Germany) systems, which have been investigated for their ability to facilitate gutta-percha removal and canal reinstrumentation [7, 8]. While instrument design is a primary variable, the final apical dimensions required to ensure adequate cleanliness in complex, curved anatomy remain poorly defined. Most previous studies have focused primarily on comparing instrument performance rather than examining how different retreatment philosophies, such as minimally invasive versus conventional apical enlargement, influence filling removal.

Furthermore, the available evidence is methodologically heterogeneous and often based on simplified anatomical models. Many studies have been conducted in straight canals [7, 9, 10]. Even when both desobstruction and reinstrumentation were evaluated, analyses were usually restricted to a single final time point. Moreover, no previous study has used high-resolution micro–computed tomography to compare filling material removal between these retreatment systems across different apical enlargement strategies.

In contemporary endodontics, there is increasing interest in minimally invasive strategies that preserve dentin and reduce structural weakening. While this concept is well established for coronal access [11], it can also be applied to retreatment cases. Although retreatment typically involves more extensive root canal preparation, it is essential to avoid overpreparation to preserve dentin and maintain tooth integrity. The integration of advanced technologies, such as CBCT, dental operating microscopes, and improved irrigation and activation methods, could enable a balance between effective cleaning during retreatment and the preservation of structural integrity. However, it remains unclear whether smaller apical enlargement compromises filling removal, particularly in severely curved canals. Therefore, this study aimed to perform a high-resolution, three-dimensional, stage-by-stage micro-CT evaluation of filling removal in severely curved mesial roots, comparing a minimally invasive approach (D-Race/iRace with reinstrumentation 30/0.04) with a conventional (Mtwo Retreatment/Mtwo with reinstrumentation 40/0.04), both performed without solvents.

## Materials and methods

### Ethical considerations

The teeth used in this study were obtained from the Tooth Bank of Estácio de Sá University, in accordance with ethical regulations for the use of human-derived biological material. All specimens were anonymized before analysis, and informed consent for the use of extracted teeth for research purposes was obtained from the donors, according to institutional guidelines. Teeth were extracted for reasons unrelated to this investigation, such as periodontal or prosthetic indications. The study protocol was approved by the Research Ethics Committee of Estácio de Sá University (CAAE 91725218.8.0000.5284).

### Sample selection

Sample size calculation was based on data from a previous micro–CT study that evaluated the removal of root filling material during endodontic retreatment [12]. Because a paired intraroot design was adopted, where both retreatment protocols were performed within the same mesial root to control for anatomical variability, the calculation considered paired comparisons. Assuming an alpha level of 0.05 and a statistical power of 80%, the minimum required sample size was determined to be 12 canals per group. Given the possibility of loss, the sample size was expanded to 14.

From a pool of 143 extracted mandibular first molars, 14 teeth were selected. Inclusion criteria were Vertucci type IV canal configuration [13] and mesiodistal curvature (25°-40°) classified as severe according to Schneider [14]. Teeth were excluded and replaced if they presented with canals lacking independent foramina, calcifications, root resorption, root caries, or cracks. This selection was performed by inspection under a clinical microscope at different magnifications and digital radiographic evaluation in buccolingual and mesiodistal projections (Rx-RVG, Kodak 5100, Marne-la-Vallée, France).

### Root canal preparation and obturation

The crowns were partially sectioned using a diamond disc (Brasseler, Savannah, GA) mounted on a motor, 3 mm above the cementoenamel junction, exposing the pulp chamber. This procedure standardized coronal access and created a platform for establishing the working length (WL) reference point. A reference mark was created on the external root surface using a high-speed spherical bur to standardize orientation during micro-CT scanning and allow accurate canal identification. Root canals were initially negotiated with a #10 K-file (Dentsply Sirona, Ballaigues, Switzerland), advanced until its tip reached and became visible at the apical foramen under 10× magnification, thereby confirming canal patency length and the presence of two separate apical foramina.

All mesial canals were prepared by a single operator using a HyFlex EDM NiTi instrument (25/0.04) (Coltene, Altstätten, Switzerland) in continuous rotation on a VDW Silver motor (VDW, Munich, Germany) at 500 rpm and 2.5 N·cm torque. WL was established 1 mm short of the root apex. Canal patency was maintained using a size 15 K-file (Dentsply Sirona) during instrumentation. This standardization, using the commonly employed 25/0.04 dimension in primary treatment, ensured uniform canal conditions prior to the retreatment procedures.

Irrigation was performed with 2 mL of 2.5% sodium hypochlorite after each instrument removal, delivered through a 30-G NaviTip needle (Ultradent Products, South Jordan, UT) positioned 3 mm short of the WL. After instrumentation, the smear layer was removed with 2 mL of 17% EDTA for 3 min, followed by a final rinse with 3 mL of 2.5% sodium hypochlorite.

Canals were dried with paper points and obturated using the single-cone technique with HyFlex 25/0.04 gutta-percha cones (Coltene) and Sealer 26 (Dentsply Sirona). The quality of the root canal fillings was confirmed by digital radiography evaluation in buccolingual and mesiodistal projections (Rx-RVG, Kodak 5100, Marne-la-Vallée, France), and no voids were detected. The specimens were then stored at 37 °C in 100% humidity for 30 days to allow complete setting of the sealer.

Prior to retreatment, all teeth underwent an initial micro-CT scan, as described in detail below. A paired intraroot design was adopted to minimize anatomical variability by allowing both retreatment protocols to be tested within the same mesial root. In each mesial root, one canal was assigned to a minimally invasive retreatment protocol (D-Race followed by iRace to 30/0.04), whereas the other canal underwent a conventional retreatment protocol (Mtwo Retreatment followed by Mtwo to 40/0.04). Allocation was performed using a strictly alternating sequence between mesiobuccal and mesiolingual canals in consecutive teeth to ensure balanced distribution of the protocols according to canal position and reduce potential allocation bias. All instruments were used according to the manufacturers’ instructions, activated by a VDW Silver motor (VDW, Munich, Germany). Each instrument was used twice and discarded. After the first use and before the second, the instruments were inspected under magnification for signs of deformation or distortion; no visible defects were observed.

### Retreatment procedures

1. Minimally invasive retreatment protocol (*n* = 14 canals)

#### *Desobstruction with D-Race*

Cervical third filling removal was performed using the DR1 instrument (30/0.10) at 1000 rpm and 1.5 N·cm torque. The middle and apical thirds were prepared using the DR2 file (25/0.04) at 600 rpm and 1.5 N·cm torque.

#### *Reinstrumentation (final size 30/0.04)*

Reinstrumentation was performed using a sequence of iRace 15/0.06, 25/0.04, and 30/0.04 instruments at 600 rpm and 1.5 N·cm torque until WL was reached. Apical patency was verified with a size 15 K-file (Dentsply) after each instrument change.

2. Conventional Retreatment Protocol (*n* = 14 canals)

#### *Desobstruction with Mtwo Retreatment*

Initial filling removal was performed using Mtwo Retreatment instruments, beginning with the 15/0.05 file to WL, followed by the 25/0.05 file, using progressive in-and-out movements. Instruments were operated at 280 rpm with torques of 0.3 N·cm and 1.2 N·cm, respectively, according to the manufacturer’s instructions.

#### *Reinstrumentation with Mtwo (final size 40/0.04)*

Reinstrumentation was performed using the sequence 25/0.06, 30/0.05, 35/0.04, and 40/0.04 to WL at 280 rpm and torque recommended by the manufacturer. Apical patency was verified with a size 15 K-file (Dentsply) after each instrument change.

The retreatment procedures were deemed complete once the iRace 30/0.04 or Mtwo 40/0.04 instruments reached the WL, no residual filling material was observed on the instrument flutes, apical patency was confirmed with a size 15 K-file (Dentsply), and the final irrigation was performed (see below).

### Irrigation conditions

For both systems, irrigation was performed after each instrument using 2.5% NaOCl for 60 s. After reinstrumentation, the smear layer was removed with 2 mL of 17% EDTA for 3 min, followed by a final rinse with 2 mL of NaOCl. Canals were then dried with paper points. During the retreatment procedures, the irrigant was simultaneously aspirated using a cannula positioned at the coronal access.

### Micro-CT scanning and analysis

The scans were performed at three stages: (1) initial, obturated canals; (2) after desobstruction; (3) after reinstrumentation. All scans were performed using a SkyScan 1174 v2 micro-CT scanner (Bruker microCT, Kontich, Belgium) at 50 kV, 800 µA, 17-µm isotropic resolution, 1° rotation step, and 180° rotation with a 0.5-mm aluminum filter. Images were reconstructed using NRecon v1.6.9 (Bruker microCT) with standardized parameters (smoothing 5, ring artifact correction 5, beam hardening correction 50%). Filling material volume (mm³) was quantified using CTAn v1.5.4.0 (Bruker microCT) after binarization. Three-dimensional visualization was performed using CTVol v2.2.1.

### Statistical analysis

Data were analyzed using SPSS 21.0 (IBM, Armonk, NY, USA). Normality was assessed using the Kolmogorov–Smirnov and Shapiro–Wilk tests, as well as graphical analysis. Variables were expressed as mean ± standard deviation and median (minimum–maximum). Because data were non-normally distributed and paired, comparisons between groups were performed using the Wilcoxon signed-rank test. The significance level was set at 5% (*P* < 0.05).

## Results

During filling removal, one tooth was excluded due to instrument fracture (Mtwo Retreatment 25/0.05), leaving 13 mesial roots available for the final analysis.

**Fig. 1:**
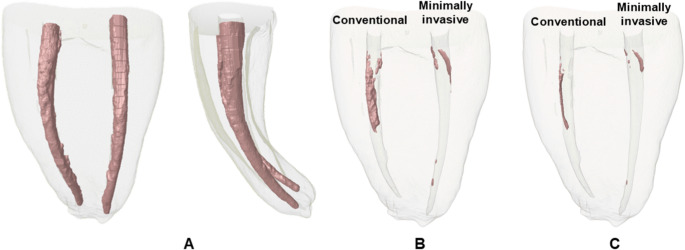
Representative three-dimensional micro-CT reconstructions illustrating the distribution of root canal filling material before and after retreatment performed through the different tested retreatment approaches. (**A**) Initial root canal obturation showing the canals completely filled (mesial and vestibular views). (**B**) Residual filling material after the desobstruction phase. (**C**) Residual filling material after the reinstrumentation phase. The pink areas represent the remaining obturation material within the root canals

Initial filling volumes were comparable between groups (*P* > 0.05). After the initial removal phase, the minimally invasive protocol (D-Race) group demonstrated a mean residual filling volume of 0.2 mm³, corresponding to approximately 95% of the original filling material removed. In contrast, the conventional protocol (Mtwo Retreatment) showed a mean residual volume of 1.1 mm³, corresponding to approximately 70% removal (Table 1; Fig. 1). This difference was statistically significant (*P* < 0.01).

Following reinstrumentation, the mean residual filling volumes were 0.1 mm³ for the minimally invasive protocol and 0.2 mm³ for the conventional protocol. The corresponding mean percentages of filling material removal were 98% and 95%, respectively (Table 1; Fig. 1). No statistically significant differences were observed between protocols at this stage (*P* > 0.05).

**Table 1:** Micro–CT quantitative volumetric analysis (mm^3^) of filling removal in minimally invasive and conventional retreatment protocols

Group	Initial filling volume		Residual volume after desobturation		Removal after desobturation (%)		Residual volume after reinstrumentation		Removal after reinstrumentation (%)	
	Mean ± SD	Median (range)	Mean ± SD	Median (range)	Mean ± SD	Median (range)	Mean ± SD	Median (range)	Mean ± SD	Median (range)
Minimally invasive (30/0.04)	3.8 ± 0.6	3.8 (2.8–4.7)	0.2 ± 0.2	0.1 (0.0–1.0)	95 ± 4.9	98.6 (86.4–100)	0.1 ± 0.2	0.0 (0.0–0.4)	98 ± 2.9	99.6 (89.0–100)
Conventional (40/0.04)	3.9 ± 0.7	3.6 (2.6–4.9)	1.1 ± 1.2	1.0 (0.0–3.7)	70 ± 32.0	80.0 (12.2–100)	0.2 ± 0.3	0.1 (0.0–1.0)	95 ± 7.2	98.0 (77.0–100)

Data are expressed as mean ± standard deviation and median (minimum–maximum). Intergroup comparisons were performed using the Wilcoxon signed-rank test (paired intraroot design). Significance level was set at α = 0.05

## Discussion

The present study compared two retreatment philosophies in severely curved mesial roots of mandibular molars: a minimally invasive protocol with limited apical enlargement (30/0.04) and a conventional protocol with greater apical enlargement (40/0.04). Mesial roots with Vertucci type IV configuration and curvature angles classified as severe were selected to enable both protocols to be applied within the same anatomical context using a paired intraroot design. This approach minimized anatomical variability and enhanced methodological standardization, thereby reducing the potential anatomical bias associated with simple randomization [15, 16]. Mandibular molars were intentionally selected because of their anatomical complexity and frequent association with post-treatment disease, making them common candidates for nonsurgical retreatment [17, 18].

Effective removal of filling material represents a critical objective in retreatment, particularly in cases of persistent intraradicular infection [2]. Residual obturation material may shield microorganisms from irrigants and mechanical instrumentation, thereby contributing to sustained periapical inflammation [3, 19, 20]. Consequently, identifying retreatment strategies that achieve substantial filling removal while preserving radicular dentin is clinically relevant.

Solvents were deliberately excluded to isolate the mechanical performance of the tested systems. Rotary instrumentation generates frictional heat that softens gutta-percha, facilitating its mechanical engagement and removal. In contrast, solvents may produce a softened layer that adheres to canal walls and potentially interferes with the complete elimination of filling remnants [4, 21]. By eliminating chemical adjuncts, the present design allowed a direct comparison of the mechanical efficiency of the minimally invasive and conventional protocols.

The systems were selected because these systems differ substantially in instrument design, kinematics, taper progression, and shaping philosophy, which may directly influence retreatment efficiency, dentin removal, and apical enlargement [7, 8]. The D-Race/iRaCe system is characterized by higher rotational speeds, an active cutting tip in the DR1 instrument, and a more conservative reinstrumentation approach associated with smaller final apical enlargement. In contrast, the Mtwo Retreatment/Mtwo system presents an S-shaped cross-sectional design with two cutting edges and is commonly associated with a more conventional shaping philosophy that emphasizes greater apical enlargement [7, 8]. These differences may affect the thermoplasticization and coronal displacement of gutta-percha, cutting efficiency, canal transportation, and the amount of radicular dentin removed during retreatment [22].

Although previous investigations have evaluated the D-Race/Race and Mtwo Retreatment/Mtwo systems, most studies were conducted in single-rooted premolars and relied on two-dimensional or low-resolution assessment methods, such as stereomicroscopy, clearing techniques, or digital image analysis (see Table 2), which limit the precision of residual material quantification and restrict extrapolation of findings to more complex root canal anatomies. To ensure methodological rigor and comparability, canals were standardized with respect to initial filling quality and volume. Retreatment completion was defined as the absence of visible filling material on the instrument flutes, a criterion frequently used in experimental retreatment studies to ensure procedural standardization. Additional radiographic reassessment or repeated removal attempts were intentionally avoided, as these could introduce operator-dependent bias, increase procedural variability and work time, and compromise direct comparison of the intrinsic efficacy of the tested protocols. Although the present experimental design differs from clinical reality, where retreatment cases often involve poorly executed obturations and clinicians may perform additional attempts to reduce residual filling material [23], the controlled conditions allowed for a more precise comparison between the two tested protocols and allow comparisons with previous studies.

**Table 2:** Synthesis of studies comparing the filling material removal with Mtwo Retreatment and D-Race

Study	Tooth type	Sample size/group	Anatomy & curvature	Curvature	Evaluation method & precision	Procedural phases evaluated	Key scientific contribution	System efficacy results
Present study	Mandibular 1st Molars (Mesial Roots)	13	Vertucci Type IV	25°-40° (MD)	Micro-CT (17-µm Isotropic Resolution)	Intragroup 3-Stage Sequential evaluation: Obturation, Deobstruction, Reinstrumentation	Isolated the reinstrumentation phase as critical for final cleanliness in complex anatomy	Initial Phase: D-Race was significantly more efficient than Mtwo. Final Phase: Systems were comparable after reinstrumentation
Patil et al. [10]	Cleared TransparentMandibular Premolars	20	Single Straight Canal	None	Stereomicroscope (8x)	Final assessment only: desobstruction	Comparative efficacy of D-Race, ProTaper, and Mtwo in straight anatomy	D-Race was significantly more effective than Mtwo
Baig et al. [9]	Mandibular Premolars	20	Single Straight Canal	None	Stereomicroscope (20x); Scoring System (1–4)	Final assessment only: desobstruction + reinstrumentation	Compared D-Race, Mtwo, and H-files with/without orange oil solvent	D-Race was significantly more effective than Mtwo
Bhagavaldas et al. [24]	Mandibular Premolars	24	Almost straight canals	N/a	Stereomicroscope (20x); 2D AutoCAD measurements	Final assessment only: desobstruction + reinstrumentation	Compared D-Race and Mtwo with/without Endosolv R solvent	D-Race was significantly more effective than Mtwo
Akhavan et al. [8]	Mandibular 1st Molars (Distal Roots)	12	Not specific	< 20° (MD)	Stereomicroscope (16x); 2D Area Processing	Final assessment only: desobstruction + reinstrumentation	Compared Mtwo and D-Race, but limited by 2D analysis and low curvature	Final: Mtwo and D-Race performed similarly with no significant differences
Marques da Silva et al. [7]	Premolars	15	Straight single canals	None	Scanner (1200 dpi); 2D AutoCAD measurements	Intergroup evaluation: Desobstruction and Desobstruction + Reinstrumentation	Evaluated if larger apical diameters improved cleanliness in straight canals	Mtwo and D-Race performed similarly with no significant differences

During the initial phase of filling removal, the minimally invasive protocol demonstrated significantly greater efficiency, achieving approximately 95% removal of the original obturation material compared with approximately 70% for the conventional protocol. Direct comparison with previous studies is limited by their focus on single final time points, lack of isolation of the initial desobstruction phase, and frequent absence of mandibular molars (see Table 2). The observed difference between the D-Race and Mtwo Retreatment instruments may be related to specific design characteristics and operational parameters, including higher rotational speeds that enhance thermoplasticization and facilitate coronal displacement of gutta-percha [24, 25].

Following reinstrumentation, both protocols demonstrated further reduction of residual filling material and achieved comparable outcomes despite the difference in final instrument sizes (30/0.04 vs. 40/0.04). This finding suggests that the mechanical debridement performed during the reinstrumentation phase may act as a procedural “equalizer,” compensating for initial differences in system efficiency. 

Although the initial sample size calculation indicated a minimum of 12 specimens, a post hoc power analysis confirmed that the final sample size (*n* = 13) provided sufficient sensitivity to detect volumetric differences greater than 0.15 mm³. During the reinstrumentation phase, a small-to-moderate effect size (Cohen’s d = 0.48) was observed, reflecting the minimal difference in residual filling volume between groups (0.05 mm³ vs. 0.17 mm³). Since both protocols achieved > 95% removal of the original obturation material, the absence of a statistically significant difference is more likely indicative of comparable mechanical effectiveness than insufficient statistical power. Notably, this outcome favors the minimally invasive protocol (D-Race + iRaCe to 30/0.04). Collectively, these findings suggest that greater apical enlargement does not necessarily result in superior removal of obturation remnants in severely curved canals.

From a clinical perspective, this observation is highly relevant. Larger apical preparations have traditionally been advocated to enhance disinfection; however, they may also result in greater dentin removal and potential structural weakening [26, 27]. Conversely, reduced apical enlargement may preserve tooth structure but could potentially compromise disinfection in anatomically complex areas. The present results suggest that, even in severely curved canals, a minimally invasive retreatment approach can achieve comparable filling removal while potentially preserving more radicular dentin. Nevertheless, further studies are needed to compare the antimicrobial efficacy of both protocols.

A thorough understanding of the relationship between dentin thickness and the selection of NiTi instruments is essential for successful root canal treatment. Instrument selection should be tailored to root canal dimensions while considering the risks of perforation and instrument fracture [28]. Cone-beam computed tomography and micro-CT studies have demonstrated substantial variability in dentin thickness in mandibular molars, with critically thin areas frequently located in the mesial root danger zones, where excessive enlargement may compromise structural integrity [29]. The average dentin thickness in the apical third of the root canal is 1 mm, decreasing to 0.77 mm near the apex [29]. Although these values may be considered safe for primary treatment, they become more critical during retreatment, particularly in previously enlarged and adequately prepared canals, which are more susceptible to over-preparation. Furthermore, micro-CT analyses comparing apical canal dimensions of mandibular molar mesial roots with commercially available NiTi instruments emphasize the importance of matching instrument size and taper to anatomical constraints to balance shaping efficacy with the preservation of radicular dentin [30, 31]. Similar studies could establish standardized instrument dimensions tailored for retreatment, thereby optimizing this clinical balance.

Previous investigations have reported that both rotary retreatment systems may induce dentinal defects, including microcracks and craze lines, during filling removal [32, 33]. Although the D-Race system incorporates an active cutting tip on the DR1 instrument to facilitate initial penetration, an element that could theoretically increase localized stress, no instrument separation, significant dentinal damage, or procedural errors were observed during either desobstruction or reinstrumentation with this instrument in the present study. In addition, available evidence indicates similar apical transportation in both mesiodistal and buccolingual directions, further supporting the mechanical safety of these retreatment instruments in curved mandibular molars [34].

## Conclusion

The minimally invasive protocol demonstrated greater efficiency in the initial removal of obturation material. Nevertheless, after final reinstrumentation, both approaches achieved comparable outcomes, leaving < 5% residual material. These findings suggest that conservative apical enlargement to size 30/0.04 may be as effective as larger preparations (size 40/0.04) for removing filling material in severely curved canals while potentially better preserving radicular dentin.

## Data Availability

No datasets were generated or analysed during the current study.
